# Gastroesophageal reflux disease in our asthma patients: the presence of dysphagia can influence pulmonary function

**DOI:** 10.1186/2049-6958-7-53

**Published:** 2012-12-17

**Authors:** Gulfidan Aras, Dilek Kanmaz, Figen Kadakal, Sevim Purisa, Kenan Sonmez, Esin Tuncay, Arzu Ozdemir

**Affiliations:** 1 Yedikule Chest Disease and Surgery Education and Research Hospital, Istanbul, Turkey; 2 Medical Faculty of Istanbul University, Department of Biostatistics, Istanbul, Turkey; 3 Çorlu Reyap Hospital, Department of Cardiology, Tekirdağ, Turkey; 4 Taksim Education and Research Hospital, Istanbul, Turkey

**Keywords:** Asthma, Dysphagia, Gastroesophageal reflux disease, Lung function

## Abstract

**Background:**

The prevalence of Gastroesophageal Reflux Disease (GERD) in Turkey is reported as 11.6%. Studies of pulmonary function in asthmatics have demonstrated a correlation between lung resistance and the occurrence of spontaneous gastroesophageal reflux. Few studies have included measures of lung diffusing capacity for carbon monoxide. The aim of this study is to assess whether asthma patients had worse lung function and gas diffusion according to diversity of GERD symptoms they concurrently experienced. The secondary aim of the study is to determine the frequency and different faces of GERD in our asthma patients compared to healthy controls.

**Methods:**

Sixty consecutive asthma patients evaluatd at the pulmonary specialty outpatient clinic were included in the study. The control group included 60 healthy volunteers who had normal pulmonary function and routine laboratory tests. A modified version of a self-reported questionnaire developed by Locke and associates at the Mayo Clinic was conducted face-to-face with consecutive asthma patients and control subjects. Pulmonary function measurements were taken using spirometry. DLCO (mL/dk/mmHg) and DLCO/VA (DLCO adjusted according to alveolar volume) were measured using a single-breath technique. Statistical analyses were performed using the SPSS 17.0 statistical software.

**Results:**

DLCO and DLCO/VA were significantly lower in asthma patients who had dysphagia symptoms. Frequent and significant acid regurgitations were seen in 28.33% (n = 17) of patients in the study group and 6.7% (n = 4) of patients in the control group. Severe, troublesome heartburn symptoms were reported by 28.2% (n = 17) of patients in the study group and 16.7% (n = 10) of subjects in the control group. Dysphagia was detected in 38.3% (n = 23) of all asthma cases and in 1.7% (n = 1) of the subjects in the control group.

**Conclusions:**

There were many faces of gastroesophageal reflux disease in our asthmatic patients. Dysphagia was the only GERD symptom influencing on pulmonary function tests, while gastroesophageal reflux symptoms and nocturnal awakening attacks were common in this study.

## Background

Gastroesophageal reflux disease (GERD) is rather common and its prevalence in Turkey is 11.6%. GERD is frequent in adult patients with asthma and the reported prevalence ranges from 32 to 82% [1-3].

Bor et al. in a study in Turkey reported in patients with asthma and in controls that the prevalence of GERD (heartburn/regurgitation once a week or more) was 25.4% and 19.4% and that of occasional symptoms (less than weekly) 21.2% and 27.0%, respectively [4].

Extraesophageal reflux disease is commonly seen at gastroenterology outpatient clinics as well as ear, nose and throat, allergy and asthma clinics. The reflux of acid and pepsin may affect the respiratory tract and cause respiratory problems such as asthma, pneumonia and interstitial fibrosis. Chronic cough, chronic laryngitis and asthma are significantly associated with GERD [5].There is a cause and effect relationship between asthma and gastroesophageal reflux which can turn into a vicious cycle. Reflux may precipitate asthma either via a vagal reflex initiated by gastric fluid in the esophagus or by micro-aspiration of gastric content into the trachea. Inversely, asthma may promote reflux due to the increased gradients pressure between the abdominal cavity and thorax, outgoing the lower esophageal sphincter pressure barrier [6-8]. Experimental evidence in both animals and humans has demonstrated reflex stimulation of bronchospasm and cough as a response to esophageal acidification.

Animal studies also have demonstrated the development of laryngeal ulceration and severe bronchospasm as a result of the direct application of acid into the larynx or acid inhalation into the airways [9-11]. Some studies of pulmonary function in asthmatics have demonstrated a correlation between lung resistance and occurrence of spontaneous gastroesophageal reflux [12]. In a review by Field *at al.* there was no objective improvement in pulmonary function attributable to GERD medical therapy. The discomfort associated with GERD can cause reflux-associated respiratory symptoms even when the pulmonary function is normal [9].

However, few studies have included measures of the lung diffusing capacity for carbon monoxide. The only study on the subject has found that severe GERD is associated with an impairment of gas exchange [13]. The aim of this study is to assess whether asthma patients have worse lung function and gas diffusion according to the diversity of GERD symptoms they concurrently experience. The secondary aim of the study is to determine the frequency of GERD in asthma patients compared to healthy controls.

## Methods

Sixty consecutive asthma patients participated in the study, and they had been seen at the pulmonary specialty outpatient clinic at the Taksim Training and Research Hospital and the Yedikule Chest Disease Hospital between January 2008 and January 2009. Several inclusion parameters were used to determine eligibility for the study. Eligible patients had been diagnosed with asthma according to the guidelines of the Global Strategy for Asthma Management (GINA) at least 3 years prior to entry into the study. The asthma severity of the patients without exacerbation was defined in this study as intermittent, mild persistent, moderate persistent, and severe persistent according to GINA [14]. None of the patients were experiencing an asthma exacerbation. All patients were receiving inhaled steroids, beta agonists or leukotriene antagonists at an appropriate level for the severity of their disease. None of the patients had been previously diagnosed with any other obstructive or restrictive chronic pulmonary diseases and patients who had pulmonary abnormalities on chest radiographs were excluded. Additionally, patients with known esophageal disease such as cancer, achalasia, stricture, active peptic ulcer disease, Zollinger-Ellison syndrome, and scleroderma as well as patients who were currently abusing alcohol (more than three alcoholic drinks a day) were excluded. This was a the symptom based study, thus an invasive diagnostic toollike the esophago-gastroduodenoscopy was not used.

### Control group

The healthy control group consisted of 60 age-matched voluntary persons who had a normal pulmonary function and routine laboratory tests. These subjects denied having any respiratory symptoms such as dyspnea or chronic sputum production and did not have a previous diagnosis of asthma or COPD or any other respiratory illness. The above-mentioned exclusion causes were also evaluated in the control group.

### Protocol

A modified version of a self-reported questionnaire developed by Locke and associates at the Mayo Clinic [15] was conducted face-to-face with consecutive asthma patients and control subjects. We established the feasibility, reproducibility, reliability and validity of the Turkish version for examining four symptoms in detail: heartburn (which was defined as a burning sensation in the retrosternal area), acid regurgitation (which was defined as the perception of the flow of refluxed gastric content into the mouth or hypopharynx), dysphagia (which was defined as a perceived impairment of the passage of food from the mouth into the stomach) and chronic cough. The first question for each symptom served as a branch point- subjects who indicated “no” proceeded to the next symptom. The next two questions for each symptom addressed the frequency (once in a month, more than once in a month, once in a week, more than once in a week and daily symptoms) and the severity of the symptoms in the last year. Further questions assessed specific attributes of each symptom. Other questions assessed the effect of both heartburn and acid regurgitation on shortness of breath, cough, wheezing, chest pain, globus and dysphonia. The remainder of the questions assessed the patient’s demographic data, smoking history and use of over-the-counter antacids and prescription anti-reflux medications. In general, the questionnaires were completed in < 30 minutes by asthma patients and controls. This study was approved by our hospital ethics committee and written informed consent was obtained from all participants in the study.

### Lung function

All FVC (forced vital capacity), FEV1 (forced expiratory capacity) and PEF (peak expiratory flow) measurements were taken using spirometry (PC using the Ocean Winspiro program, Spirolab, MIR-Medical International research, Italia). DLCO (mL/dk/mmHg) and DLCO/VA (DLCO adjusted according to alveolar volume) were measured using a single-breath technique (model 1070; Medical Graphics; St. Paul, MN). The hemoglobin level was known at the time of testing. The DLCO was routinely adjusted for the hemoglobin if the value was outside of the normal range. When analyzing the pulmonary function data, the asthma patients with gastroesophageal reflux symptoms were compared to patients without symptoms. Each of the GER symptoms was separately evaluated.

### Statistical analysis

The reliability of the questionnaire was calculated for dichotomous choices using kappa statistics (test-retest). Kappa values were between 0.69–1. The questions were written using a Likert-type scale and were analyzed with the Spearman correlation (rs) test (rs values were between 0.67–0.95). The internal consistency of measures with dichomotous choices was evaluated by the Kuder-Richardson Formula 20 (KR–20) and the value obtained was 0.89. At the same time, the Cronbach α was used to assess internal consistency for measures with non-dichomotous choices and was calculated to be 0.85. The normality of the distribution was assessed using the Kolmogorov Smirnov test and the Shapiro-Wilk test by drawing histograms. The data were presented as mean ± SD, median(min-max), frequency and percentage. The variables in each group were compared using a Mann–Whitney U-test. The nominal variable values were evaluated using the Chi-square test, Yates arranged Chi-square test and Fisher exact test. Analyses were performed using the SPSS 17.0 statistical software. The statistical significance value was set at p < 0.05.

## Results

**Figure 1:**
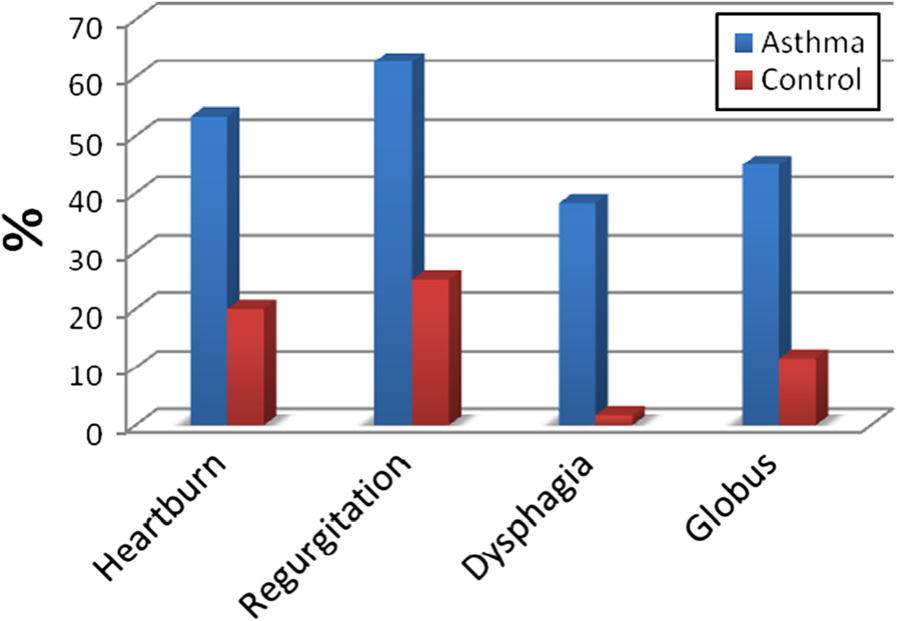
Increased frequency of gastroesophageal reflux symptoms.

**Figure 2:**
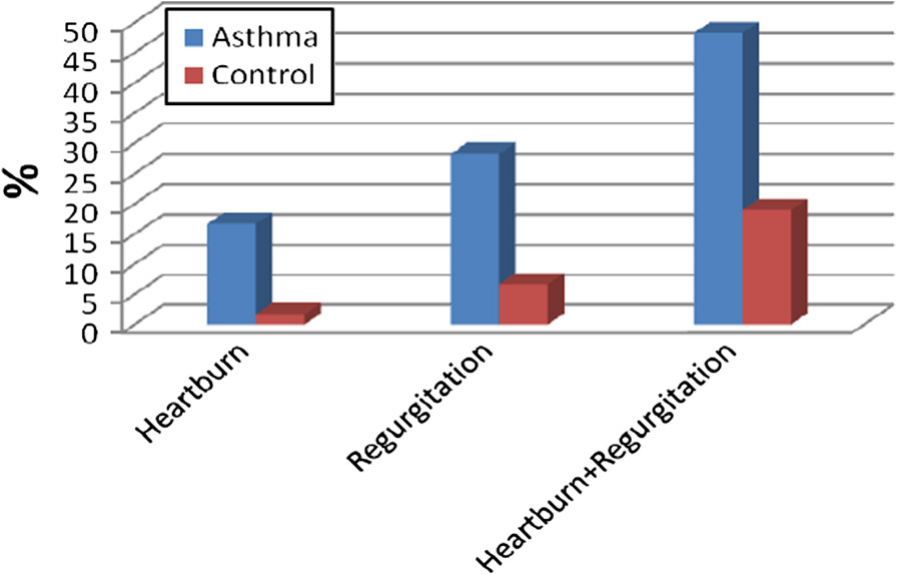
Significant and frequent gastroesophageal reflux symptoms (several times a week or everyday) and presence of heartburn with regurgitation.

The male to female ratio of the asthmatic patients was 26/24, while it was 24/26 in the control group. There was no statistically significant difference between these two groups with regard to sex (p = 0.14). The mean age of the study group was 44.85 ± 14.25 years, the mean age of the control group was 41.48 ± 13.90 years(p > 0.05) (Table 1). According to the GINA classification, 25 out of 60 patients had intermittent asthma (FEV1 and PEF ≥ 80%, predicted and variability *<*20% – step 1), 8 had mild persistent asthma (nocturnal symptoms, variability 20–30% – step 2), 21 had moderate persistent asthma (daily symptoms FEV1 and PEF 60–80%. variability ≥ 30%– step 3) and 6 had severe persistent asthma (daily symptoms, FEV1 and PEF ≤ 60%, variabi lity *>* 30% – step 4). None of the asthmatic patients was current smoker, however 10.3% (n = 6) were ex-smokers and 10.0% (n = 6) of the healthy controls were current smokers. No subjects in either group drank alcohol. Patients had not regularly used anti-reflux therapy. However, 8 had used proton-pomp inhibitors several times(13.3%). The frequency of any heartburn in the asthma cases was 53.3% (n = 32) and in the control group it was 20% (n = 12) (p = 0.001) (Figure 1). The incidence of frequent and significant heartburn (occurring daily or at least a few times a week) was 16.7% (n = 10) in the study group and 1.7% (n = 1) in the control group (p = 0.05) (Figure 2). Severe, troublesome heartburn symptoms were reported by 28.2% (n = 17) of patients in the study group and 16.7% (n = 10) of control subjects. The frequency of regurgitation in asthma patients was 62.7% (n = 37), but only 25% (n = 15) in the control group (p = 0.001) (Figure 1). Frequent and significant regurgitation was seen in 28.33% (n = 17) of patients in the study group and 6.7% (n = 4) of control subjects (Figure 2). Severe, troublesome regurgitation (as reported by the patients) was seen in 27% (n = 10) of the asthmatics with regurgitation, and 6.7% (n = 1) of subjects in the control group. Both heartburn and regurgitation were experienced by 48.3% (n = 29) of asthmatics and 19.3% (n = 11) of controls (p = 0.001) (Figure 2). No significant association was seen between heartburn, symptoms of regurgitation and a limitation of daily activities in either the study or the control group (p = 0.07). Dysphagia was detected in 38.3% (n = 23) of asthmatics and in 1.7% (n = 1) of controls (p < 0.001) (Figure 1). When patients were questioned in detail, dysphagia was reported intermittently and more often occurred for solid foods than for liquids. The dysphagia symptoms experienced by the patients did not progress. Some patients had a hard time defining the difference between dysphagia and difficulty swallowing. Frequent and significant dysphagia was found in 16.7% (n = 10) of asthmatics. Dysphonia was seen in 78.3% (n = 47), globus in 45% (n = 27) of asthmatics and 10% (n = 6) and 11.6% (n = 7) of controls, respectively (p < 0.01) (Figure 1). Chest pain was seen in 52.5% (n = 31) of asthma patients and in only 15% (n = 9) of the control subjects (p = 0.001). After a detailed history was obtained from patients with chest pain and the cardiologic workup was done, 15% (n = 9) of asthma patients had findings of ischemia (8.3% (n = 5) and hypertension (6.7% (n = 4). These findings were detected in 1.7% (n = 1) of controls (p = 0.01). Seventy percent (n = 42) of asthmatics initially presented to the hospital with chronic cough and dyspnea. Gastroesophageal reflux symptoms were the initial presentation in only 6.8% (n = 4) of the asthmatics patients. None of the patients had undiagnosed or previously known hiatal hernia or esophageal disease. There was no significant correlation between the presence of regurgitation symptoms and the clinical severity of asthma (p = 0.79 and p = 0.73, respectively). Similarly, there was no correlation between heartburn and regurgitation and severe cough (p =0.163 and p = 0.14, respectively). Nevertheless, there was a significant correlation between the presence of regurgitation and waking up at night with dyspnea in asthmatic patients (p = 0.005). When asthma patients with and without heartburn symptoms were compared, there was no difference in pulmonary function test parameters such as FEV1,FVC, FEV1/FVC, PEF and pulmonary diffusion tests parameters such as DLCO, DLCO/VA (Table 2). The presence of heartburn and regurgitation did not affect pulmonary function. However, when patients with and without dysphagia symptoms were compared, the pulmonary diffusion test parameters such as DLCO, DLCO/VA were significantly lower in patients who had dysphagia (p = 0.008, p = 0.02, respectively). Similarly, pulmonary function test parameters such as FEV1, FVC, PEF were lower in asthma patients with dysphagia (p = 0.044, p = 0.049, p = 0.019) (Table 3).

**Table 1:** Demographics of patients with asthma and control subjects

**Variables**		**Asthma Patients (n = 60)**	**Control Subjects (n = 60)**	**p**
Gender	F n(%)	34 (56.7)	26 (43.3)	0.14
	M n(%)	26 (43.3)	34 (56.7)	
Age, yr				
mean ± SD, median (min.-max.)		44.85 ± 14.25, 44(17.80)	41.48 ± 13.90 (18-72)	p > 0.05
BMI, kg/m^2^				
mean ± SD, median (min.-max.)		25.93 ± 5.07, 25.9(13-42)	24.93 ± 4.40, 24.8(17.7-33.6)	p > 0.05

**Table 2:** Spirometry and lung diffusion test measurements comparing patients with and without heartburn

	***Asthma Patients***		
	**HB (n = 32)**	**non-HB (n = 28)**	**p**
Age mean ± SD	44.69 ± 12.1	45.04 ± 16.6	0.93
BMI mean ± SD	27.05 ± 5.22	26.8 ± 5	0.85
FEV1 L mean ± SD	2.20 ± 0.80	2.08 ± 0.83	0.561
FEV1% of predicted mean ± SD	73.25 ± 21.72	69.32 ± 20.60	0.475
FVC.L mean ± SD	3.01 ± 1.11	2.91 ± 0.89	0.703
FVC% of predicted mean ± SD	82.40 ± 22.11	82.54 ± 16.04	0.977
FEV1 FVC% mean ± SD median (min.-max.)	3.02 ± 10.28 73.9(35-88)	70.53 ± 12.32 71.7(48-94)	0.405
DLCO mean ± SD	26.91 ± 8.19	26.14 ± 8.06	0.717
DLCO% mean ± SD	101.31 ± 20.10	96.71 ± 15.37	0.321

**Table 3:** Spirometry and diffusion capacity of the lung for carbon monoxide in patients with and without dysphagia

**PFT Results in Asthma Group**			
**(mean ± SD)**	**Dysphagia (n = 23)**	**non-Dysphagia (n = 37)**	**p value**
Age, yr	45.3 ± 14.7	44.57 ± 14.16	0.85
BMI	26.44 ± 6.28	27.24 ± 4.27	0.56
FEV1.L	1.89 ± 0.68	2.30 ± 0.86	0.044
FEV1% of predicted	68.69 ± 23.17	73.10 ± 19.88	0.454
FVC.L	2.66 ± 0.82	3.15 ± 1.07	0.049
FVC% of predicted	81.69 ± 20.74	83.08 ± 18.71	0.766
FEV1.FVC.%	70.63 ± 13.48	72.55 ± 9.85	0.564
DLCO	23.38 ± 6.19	28.54 ± 8.54	0.009
DLCO%	92.00 ± 13.59	103.62 ± 19.17	0.008
DLCO/VA	4.96 ± 0.81	6.30 ± 4.78	0.0103
DLCO/VA	98.66 ± 16.19	114.67 ± 19.77	0.002

## Discussion

The main findings of this study are the changes of pulmonary function in patients with dysphagia. In fact, in this study pulmonary diffusion test parameters such as DL_CO_, DL_CO_/VA were significantly lower in patients with dysphagia compared to those without dysphagia. Pulmonary function test parameters such as FEV_1_, FVC, PEF, were lower in asthma patients with dysphagia. Previous studies in the literature have already compared pulmonary function test results in patients with and without GERD, while no study has compared the pulmonary function test results according to diversity of GERD symptoms.

To the best of our knowledge there are in literature no studies other than that done by Schachteret al. in which the pulmonary diffusion capacity was measured in patients with GERD [13]. That study was performed in a group composed of obese people with severe GERD symptoms and a low diffusion capacity was found in those patients. There is no difference in the prevalence of asthma and/or airway obstruction in patients with and without GERD. In our study we did not find any changes in the diffusion capacity of patients who had heartburn/regurgitation. In Tan et al. study, GERD has not been shown to worsen lung function or bronchial reactivity consistently [16]. Field et al. determined increased minute ventilation without changing lung function in non-asthmatic patients exposed to experimental acid perfusion [17]. However, patients with dysphagia, had changes in pulmonary diffusion parameters in this study. The carbon monoxide diffusion test has been previously found to be either normal or even high in asthmatic patients [18]. The spectrum of reflux disease spans from non-erosive reflux disease to esophageal complications such as esophagitis, hemorrhage, stricture, Barrett’s esophagus and esophageal carcinoma. Prolonged exposure of the esophagus to gastric content can cause dyphagia and this can suggest that undesirable changes are likely occurring in the lung and esophagus. In a study of patients with idiopathic pulmonary fibrosis, 83% presented with abnormal distal/proximal esophageal acid exposure [19].

GERD can cause permanent histopathological changes in asthma, progression to fibrosis, and decrease in the response rate to treatment through direct irritation, hypersensitivity of the airway , and a neural reflex. An asthma exacerbation results in negative intrathoracic pressure, which may cause reflux, and medications used to treat asthma (theophylline, beta-agonists, steroids) can on turn reduce the lower esophageal sphincter pressure [20-22]. We did not find any study that showed the relationship with pulmonary fibrosis and dysphagia in asthma patients. However, Savarino et al. study explored the association of GERD and pulmonary fibrosis in patients with scleroderma with and without pulmonary fibrosis. Their findings suggested that patients with cutaneous systemic sclerosis and pulmonary fibrosis have more extensive esophageal involvement, leading to more severe GERD. Low LES (lower esophageal sphincter) pressure and smooth muscle dysfunction (i.e., uncoordinated pressure waves and reduced or absent peristalsis) predisposed to GERD in these patients [23,24]. Their experiments can be model to explain the coincidence of dysphagia and pulmonary fibrosis [23-25]. The result of Masato et al. study supported that lung inflation modulated the swallowing reflex [26].The observed impaired breathing and swallowing patterns in the patients with COPD suggests a possible explanation for the development of dysphagia [27-29]. Our study was limited to prove the progression to pulmonary fibrosis or lung inflation with radiologic tools in asthmatic patients with dysphagia. Recurrent airway inflammation and aberrant remodeling is thought to be the basis of the fibrotic response seen in patients with asthma. The numbers of fibrocytes were significantly increased in the *lamina propria* and in the peripheral blood of patients with severe asthma [29-31]. Both the asthma itself and accompaniment of GERD may predispose to fibrosis. In this study the incidence of dysphagia was similar to that reported by Vakil et al., that is 37% in patients with erosive esophagitis [32]. In another study on asthma patients, we detected dysphagia in 26.7% of patients with esophagitis [33]. When patients were questioned in detail, dysphagia was noted to be intermittent and non-progressive. Non-troublesome and non-progressive dysphagia is common in GERD. Non-progressive dysphagia can resolve in most patients. In this study, the incidence of frequent and significant dysphagia was 16.7%. However, none of the patients had progressive dysphagia. In patients with troublesome and frequent dysphagia the extent to which the dysphagia progresses should always be determined. Progressive dysphagia is an alarm symptom that warrants further examination for peptic stricture and upper gastrointestinal malignancy [34].

According to the Montreal definition and classification, heartburn and/or regurgitation are typical gastroesophageal reflux symptoms [12]. In this study the incidence of heartburn and acid regurgitation symptoms in asthma patients were consistent with the literature [1-3]. Frequent acid regurgitation was seen in 28.3% of asthma patients. The incidence of acid regurgitation detected in asthma patients in this study was similar to the findings of Boret al*.* (25.4% of asthmatic subjects had heartburn/regurgitation once a week or more) [4]. When questioning patients, it is important to make sure that patients understand the meaning of each symptom [5]. While severe heartburn and regurgitation were present in 28.2% and 27% respectively of the studied patients, these percentages reflect the personal perception of the severity of symptoms and may be subject to personal and individual differences. Moreover, none of our patients had reflux symptoms that limited their daily activities. Heartburn/regurgitation symptoms coexisted in 48.3% of asthma patients. In the study by Klauseret al. that examined a patients’ population referred for pH monitoring, a sensitivity of 78% and a specificity of 60% for heartburn were reported [35]. The study by Moayyedi P. et al. showed that patients with “dominant heartburn” have a 50% chance of having GERD, as diagnosed by 24-h esophageal pH studies [36]. Furthermore, nonacid esophageal reflux (pH of > 4) has also been recognized [37,38]. A varying prevalence of non-cardiac chest pain has been reported in studies from a variety of countries. A number of studies have reported a population prevalence of chest pain of up to 25% [39]. A study in China was similar to our study with a population prevalence of chest pain of 20.6%. The authors found that GERD was present in 51% of subjects with non-cardiac chest pain [40]. GERD can cause episodes of chest pain that resemble ischemic cardiac pain without accompanying heartburn or regurgitation according to the Montreal definition and classification. Esophageal motor disorders can also cause chest pain by a mechanism separate from gastroesophageal reflux [5]. Furthermore, non-respiratory symptoms such as chest pain are common symptoms of an asthma attack prodrome and become more frequent after the onset of the attack [41]. Future studies that investigate chest pain in the setting of GERD and asthma should take into account these contradictions. In the present study there was no significant correlation between the presence of gastroesophageal symptoms and the clinical severity of asthma. On the contrary, in some studies the severity of asthma has been associated with reflux symptoms. In fact, reflux symptoms were present in 30% of patients with mild asthma, 76% of those with moderate asthma and 70% of those with severe asthma [36]. However, other studies do not support these findings. Patients with well-controlled asthma with and without pH probe-documented acid reflux were compared in a study by Di Mango et al*.* and their results did not support the conclusion that asymptomatic reflux is associated with decreased lung function, worsen asthma control, increased airway hyperresponsiveness or increased asthma symptoms [42]. Many more studies are needed to investigate the association between reflux symptoms and the severity of asthma. However, the presence of acid regurgitation was significantly correlated with nocturnal awakening with dyspnea in this study. Compared to non-asthmatics, asthmatic patients have significantly more frequent and severe day and night GERD symptoms and significantly more pulmonary symptoms (nocturnal suffocation, cough or wheezing) often associated with GERD [43]. In a study done in Brazil, awakening from sleep was the most frequent symptom found at the onset of GERD, and preceded 38% of nocturnal GERD. Sleep stage 2 was also prevalent and preceded 36% of GER events [44].

## Conclusions

In conclusion, dysphagia was the only influential GERD symptom on pulmonary function test while gastroesophageal reflux symptoms and nocturnal awakening attacks were common in this study. Dysphagia should be an alarm sign for the pulmonologist in the same manner that it is for the gastroenterologist. A pulmonary carbon monoxide diffusion test should be used in the follow up of patients who have coexisting asthma and GERD. However, more studies that investigate this topic are needed.

## Competing interest

The authors declare that they have no competing interests.

## References

[B1] Sontag SJ O'Connell S Khandelwal S Miller T Nemchausky B Schnell TG Serlovsky R Most asthmatics have gastroesophageal reflux with or without bronchodilator therapy Gastroenterology 1990 99 613 620 2379769 10.1016/0016-5085(90)90945-w

[B2] Vincent D Cohen-Jonathan AM Leport J Merrouche M Geronimi A Pradalier A Soulé JC Gastro-oesophageal reflux prevalence and relationship with bronchial reactivity in asthma Eur Respir J 1997 10 2255 2259 10.1183/09031936.97.10102255 9387949

[B3] Field SK Underwood M Brandt R Cowie RL Prevalence of gastroesophageal reflux symptoms in asthma Chest 1996 109 316 322 10.1378/chest.109.2.316 8620699

[B4] Bor S Kitapcioglu G Solak ZA Ertilav M Erdinc M Prevalence of gastroesophageal reflux disease in patients with asthma and chronic obstructive pulmonary disease J Gastroenterol Hepatol 2010 25 2 309 313 10.1111/j.1440-1746.2009.06035.x 19817951

[B5] Vakil N Zanten SV Kahrilas P Dent J Jones R Global Consensus Group The Montreal definition and classification of gastroesophageal reflux disease: a global evidence-based consensus Am J Gastroenterol 2006 101 1900 1920 10.1111/j.1572-0241.2006.00630.x 16928254

[B6] Sontag SJ Why do the published data fail to clarify the relationship between gastroesophageal reflux and asthma? Am J Med 2000 108 Suppl 4a 159S 169S 10718471 10.1016/s0002-9343(99)00357-5

[B7] Harding SM Gastroesophageal reflux, asthma, and mechanisms of interaction Am J Med 2001 111 Suppl 8A 8S 12S 11749917 10.1016/s0002-9343(01)00817-8

[B8] Moote DW Lloyd DA McCourtie DR Wells GA Increase in gastroesophageal reflux during methacholine-induced bronchospasm J Allergy Clin Immunol 1986 78 4 Pt 1 619 623 3534050 10.1016/0091-6749(86)90079-5

[B9] Field SK Evans JA Price LM The effects of acid perfusion of the esophagus on ventilation and respiratory sensation Am J Respir Crit Care Med 1998 157 1058 1062 9563719 10.1164/ajrccm.157.4.9707094

[B10] Adhami T Godblum JR Richter JE Vaezi MF The role of gastric and duodenal agents in laryngeal injury: an experimental canine model Am J Gastroenterol 2004 99 2098 2106 10.1111/j.1572-0241.2004.40170.x 15554987

[B11] Tuchman DN Boyle JT Pack AI Scwartz J Kokonos M Spitzer AR Cohen S Comparision of airway responses following tracheal or esophageal acidification in the cat Gastroenterology 1984 87 872 881 6468875

[B12] Cuttitta G Cibella F Visconti A Scichilone N Bellia V Bonsignore G Spontaneous gastro-esophageal reflux and airway patency during the night in adult asthmatics Am J Respir Crit Care Med 2000 161 177 181 10619817 10.1164/ajrccm.161.1.9808014

[B13] Schachter LM Dixon J Pierce RJ O'Brien P Severe gastroesophageal reflux is associated with reduced carbon monoxide diffusing capacity Chest 2003 123 1932 1938 10.1378/chest.123.6.1932 12796170

[B14] National Institutes of Health National heart. Lung. And blood institute global ınitiative for asthma Global strategy for asthma management and prevention NHIBI/WHO workshop report 2002 Revised 2006

[B15] Locke GR Talley NJ Weaver AL Zinsmeister AR A new questionnaire for gastroesophageal reflux disease Mayo Clin Proc 1994 69 6 539 547 10.1016/S0025-6196(12)62245-9 8189759

[B16] Tan WC Martin RJ Pandey R Ballard RD Effects of spontaneous and simulated gastroesophageal reflux on sleeping asthmatics Am Rev Respir Dis 1990 141 1394 1399 2350084 10.1164/ajrccm/141.6.1394

[B17] Field SK Sutherland LR Does medical antireflux therapy improve asthma in asthmatics with gastroesophageal reflux?: a critical review of the literature Chest 1998 114 275 283 10.1378/chest.114.1.275 9674479

[B18] Saydain G Beck KC Decker PA Cowl CT Scanlon PD Clinical significance of elevated diffusing capacity Chest 2004 125 446 452 10.1378/chest.125.2.446 14769723

[B19] Raghu G Freudenerger TD Yang S Curtis JR Spada C Hayes J Sillery JK Pope CE 2nd Pellegrini CA High prevalence of abnormal acid gastro-oesophageal reflux in idiopathic pulmonary fibrosis Eur Respir J 2006 27 136 142 10.1183/09031936.06.00037005 16387946

[B20] Pacheco-Galván A Hart SP Morice AH Relationship between gastro-oesophageal reflux and airway diseases: the airway reflux paradigm Arch Bronconeumol 2011 47 195 203 21459504 10.1016/j.arbres.2011.02.001

[B21] Blondeau K Dupont LJ Mertens V Tack J Sifrim D Improved diagnosis of gastrooesophageal reflux in patients with unexplained chronic cough Aliment Pharmacol Ther 2007 25 723 732 10.1111/j.1365-2036.2007.03255.x 17311606

[B22] Patterson RN Johnston BT Ardill JE Heaney LG McGarvey LP Increased tachykinin levels in induced sputum from asthmatic and cough patients with acid reflux Thorax 2007 62 491 495 10.1136/thx.2006.063982 17251314 PMC2117233

[B23] Murphy JR McNally P Peller P Shay SS Prolonged clearance is the primary abnormal reflux parameter in patients with progressive systemic sclerosis and esophagitis Dig Dis Sci 1992 37 833 841 10.1007/BF01300380 1587187

[B24] Patti MG Debas HT Pellegrini CA Esophageal manometry and 24-hour pH monitoring in the diagnosis of pulmonary aspiration secondary to gastroesophageal reflux Am J Surg 1992 163 401 406 10.1016/0002-9610(92)90041-O 1558280

[B25] Savarino E Bazzica M Zentilin P Pohl D Parodi A Cittadini G Negrini S Indiveri F Tutuian R Savarino V Ghio M Gastroesophageal reflux and pulmonary fibrosis in scleroderma: a study using pH-impedance monitoring Am J Respir Crit Care Med 2009 179 408 413 10.1164/rccm.200808-1359OC 19096004

[B26] Masato K Shirohisono, takashınıshıno, modulation of swallowing reflex by lung volume changes Am J Respir Crit Care Med 2000 162 1855 1858 11069826 10.1164/ajrccm.162.5.2005106

[B27] Gross RD Atwood CW Jr Ross SB Olszewski JW Eichhorn KA The coordination of breathing and swallowing in chronic obstructive pulmonary disease Am J Respir Crit Care Med 2009 179 559 565 10.1164/rccm.200807-1139OC 19151193

[B28] Palmer PM Luschei ES Jaffe D McCulloch TM Contributions of individual muscles to the submental surface electromyogram during swallowing J Speech Lang Hear Res 1999 42 1378 1391 10599620 10.1044/jslhr.4206.1378

[B29] Hiss SG Strauss M Treole K Stuart A Boutilier S Swallowing apnea as a function of airway closure Dysphagia 2003 18 293 300 10.1007/s00455-003-0021-y 14571335

[B30] Schmidt M Sun G Stacey MA Mori L Mattoli S Identification of circulating fibrocytes as precursors of bronchial myofibroblasts in asthma J Immunol 2003 171 380 389 12817021 10.4049/jimmunol.171.1.380

[B31] Saunders R Siddiqui S Kaur D Doe C Sutcliffe A Hollins F Bradding P Wardlaw A Brightling CE Fibrocyte localization to the airway smooth muscle is a feature of asthma J Allergy ClinImmunol 2009 123 376 384 10.1016/j.jaci.2008.10.048 PMC3992369 19081612

[B32] Vakil NB Traxler B Levine D Dysphagia in patients with erosive esophagitis: Prevalence, severity and response to proton pump inhibitor treatment Clin Gastroenterol Hepatol 2004 2 665 668 10.1016/S1542-3565(04)00289-7 15290658

[B33] Aras G Yelken K Kanmaz D Develioglu O Mavis O Gultekin E Igdem AA Purisa S Erosive esophagitis worsens reflux signs and symptoms in asthma patients without affecting pulmonary function tests J Asthma 2010 47 1101 1105 10.3109/02770903.2010.519077 21039214

[B34] Flook N Jones R Vakil N Approach to gastroesophageal reflux disease in primary care. Putting the Montreal definition into practice Can Fam Physician 2008 54 701 705 18474703 PMC2377200

[B35] Klauser AG Schindlbeck NE Muller-Lissner SA Symptoms in gastroesophageal reflux disease Lancet 1990 335 205 208 10.1016/0140-6736(90)90287-F 1967675

[B36] Moayyedi P Axon ATR The usefulness of the likelihood ratio in the diagnosis of dyspepsia and gastro-esophageal reflux disease Am J Gastroenterol 1999 94 3122 3125 10.1111/j.1572-0241.1999.01502.x 10566701

[B37] Irwin RS Zawacki JK Wilson MM French CT Callery MP Chronic cough due to gastroesophageal reflux disease: failure to resolve despite total/near-total elimination of esophageal acid Chest 2002 121 1132 1140 26 10.1378/chest.121.4.1132 11948043

[B38] Tutuian R Mainie I Agrawal A Adams D Castell DO Nonacid reflux in patients with chronic cough on acid-suppressive therapy Chest 2006 130 386 391 10.1378/chest.130.2.386 16899836

[B39] Locke GR 3rd Talley NJ Fett SL Zinsmeister AR Melton LJ 3rd Prevalence and clinical spectrum of gastroesophageal reflux: a population based study in Olmsted county, Minnesota Gastroenterology 1997 112 1448 1456 10.1016/S0016-5085(97)70025-8 9136821

[B40] Wong WM Lam KF Cheng C Hui WM Xia HH Lai KC Hu WH Huang JQ Lam CL Chan CK Chan AO Lam SK Wong BC Population based study of non cardiac chest pain in southern Chinese: prevalence, psychosocial factors and health care utilization World J Gastroenterol 2004 10 707 712 14991943 10.3748/wjg.v10.i5.707 PMC4716914

[B41] Edmondstone WM Chest pain and non-respiratory symptoms in acute asthma Postgrad Med J 2000 76 413 414 10.1136/pmj.76.897.413 10878200 PMC1741665

[B42] DiMango E Holbrook JT Simpson E Reibman J Richter J Narula S Effects of asymptomatic proximal and distal gastroesophageal reflux on asthma severity Am J Respir Crit Care Med 2009 180 809 816 10.1164/rccm.200904-0625OC 19661245 PMC2773912

[B43] Sontag SJ O'Connell S Miller TQ Bernsen M Seidel J Asthmatics have more nocturnal gasping and reflux symptoms than nonasthmatics, and they are related to bedtime eating Am J Gastroenterol 2004 99 789 796 10.1111/j.1572-0241.2004.04141.x 15128338

[B44] Mello-Fujita L Roizenblat S Frison CR Rodrigues Junior L Garbuio S Tufik S Bittencourt LR Gastroesophageal reflux episodes in asthmatic patients and their temporal relation with sleep architecture Braz J Med Biol Res 2008 41 152 158 18297195 10.1590/s0100-879x2008000200012

